# Retrospective study about the postoperative stability of zygomaticomaxillary complex fracture

**DOI:** 10.1186/s40902-021-00311-9

**Published:** 2021-10-01

**Authors:** Seoghwan Yang, Jin-yong Cho, Woo-chul Shim, Sungbeom Kim

**Affiliations:** Dept. of Oral & Maxillofacial Surgery, Gachon University Gil Medical Center, 21, Namdong-daero 774 beon-gil, Namdong-gu, Incheon, 21565 South Korea

**Keywords:** Skull fractures, Zygomatic fractures, Fracture fixation

## Abstract

**Background:**

The aim of this study is to evaluate the postoperative stability of zygomaticomaxillary complex (ZMC) fractures according to the number of fixation sites and to investigate the direction of postoperative displacement of the unfixed part of the fractured segment.

**Methods:**

This study was retrospectively performed on 38 patients who were treated by open reduction and internal fixation of ZMC fractures and were taken postoperative computed tomography (CT) between February 2012 and July 2019. The patients were classified into 3 groups: 1-point fixation, 2-point fixation, 3-point fixation according to the number of fixations. The postoperative displacement of the fractured segment was evaluated by the superimposition between postoperative CT and follow-up CT, and the postoperative stability according to the fixation sites was investigated through the amount of postoperative displacement. In addition, it was investigated in which direction the location of the fractured segment was changed in the unfixed fractured segment according to the fixation sites.

**Results:**

The amount of postoperative displacement of the fractured segment was 0.75 ± 1.18 mm on average.

In the postoperative displacement of the distal area according to the number of fixation of the fracture, there was no statistically significant difference in the amount of displacement of the fracture (*p* = 0.574).

As for the direction of the change in the location of the fractured segment, 12 patients among 38 patients with the change in the location of the fractured segment were investigated, and the displacement in the medial direction (*n* = 11, 91.67%) was the most common in all three fixation methods.

**Conclusion:**

In patients with a ZMC fracture who were treated by open reduction and internal fixation, the number of fixations did not make the difference in the postoperative displacement of the fracture. In addition, the fractured segment mainly changes in the medial direction after surgery, and this fact can be used as a reference for the reduction direction during surgery for the stable prognosis.

## Background

The ZMC functions as a major part of the middle third of the facial bone structure and is often fractured together with other bones of the midface due to its convex shape. Since the ZMC plays an important esthetic role in the middle facial appearance, it is a structure that must be restored in the facial fracture treatment. When there is displacement of the fracture site, surgical treatment of ZMC fractures is indicated, and open reduction and internal fixation is the selected treatment methods. The number or location of the fixation and the surgical approach are determined by the type of fracture and the surgeon’s preference [1].

As for how to fix the ZMC fracture, there is still no definite consensus. The zygomatic bone has a pyramidal structure and can be fixed at 4 locations (ZMB, zygomaticomaxillary buttress; FZ, frontozygomatic suture; IOR, infraorbital rim; ZA, zygomatic arch) when fracture occurred. Surgeons have different opinions about where and how to fix it and how many places to fix it for stability [2].

A number of biophysical studies have been performed to determine the stability of ZMC fractures after open reduction surgery. It is certain that fixation at as many points as possible will improve postoperative stability. On this basis, the biggest advantage of three-point fixation is that the postoperative stability is relatively higher than that of one-point fixation or two-point fixation. Davidson et al. ZMC fractures were fixed in various ways using mini-plates, and the maximum stability against physiological loads was achieved when all three points were fixed: frontozygomatic suture, inferior orbital rim, and zygomaticomaxillary buttress [3]. Similar findings can be found in other biomechanical studies by O’Hara [4]. However, as the number of fixations increases and the procedure becomes more complex, there are disadvantages such as the formation of larger wounds, increasing the risk of wound swelling, infection, and scarring [3].

If a fixation effect of the least invasive, equivalent degree or clinically acceptable stability can be obtained with a minimum number of fixation out of the four sites, it would be an excellent fixation method. By raising these questions, many surgeons began to study the stability of the methods of fixing only two or one of the four places. However, there is still room for debate about the stability of the 1-point fixation method.

Therefore, the purpose of this study is to find the best fixation method by examining whether there is any difference in the amount of displacement of the fractured segment after surgery among 1-point fixation, two-point fixation, and three-point fixation.

## Methods

This study was performed on patients who had unilateral or bilateral ZMC fractures from February 2012 to July 2019 who visited the Gachon University Gil Hospital Dental Clinic and underwent open reduction and internal fixation in the Oral and Maxillofacial Surgery department. The subjects included patients with only 1 to 3 simple fractures in the ZMC except comminuted fractures. In addition, only patients in a systematic stable condition who could undergo open reduction and internal fixation surgery under general anesthesia were included in the study.

However, patients who were with comminuted fracture patterns and whose outpatient treatment was discontinued for follow-up after surgery were not included in the study. Regarding research ethics, this study was approved by the institutional review board of Gil Hospital, Gachon University (GDIRB2020-345).

The ZMC fractures were analyzed and the treatment plan was determined using facial computed tomography (CT) taken before the surgery. The postoperative CT was taken within a week after the surgery to confirm reduction state. Then, the fractured segment was healed and stabilized, and a follow-up CT was taken after visiting the outpatient clinic again.

Postoperative CT and follow-up CT were superimposed using the Invivo 5, version 5.3 software (Anatomage, San Jose, CA) using anatomical landmarks. During overlapping, it was performed using some reference points, like a nasion and infraorbital foramens which are expected to remain unchanged through fractures. Through this, the amount and direction of displacement of the surgically reduced fracture segments during the healing period were investigated.

**Fig. 1:**
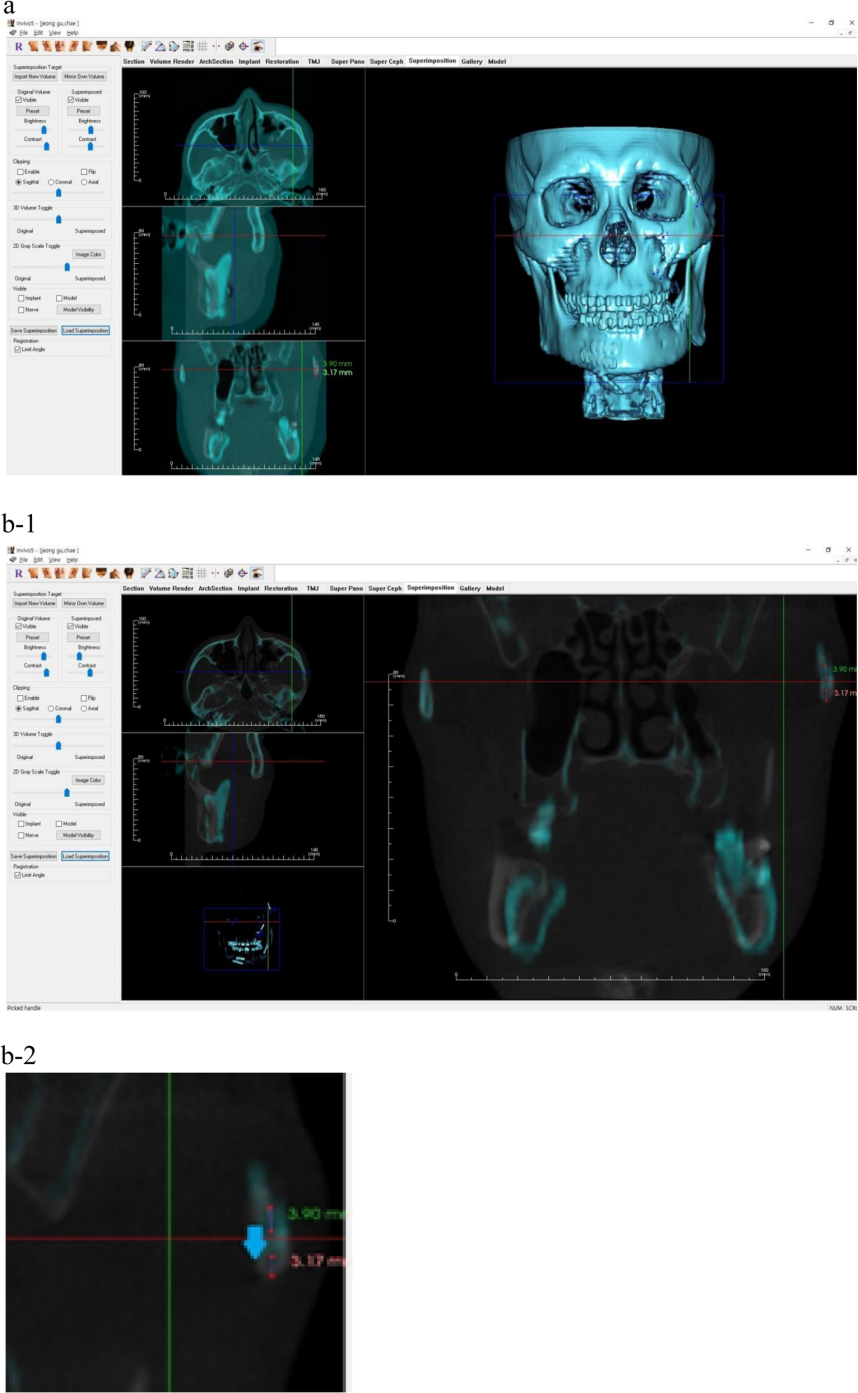
The measurement of amount and direction of displacement of the fractured segment. **a** CT images are superimposed using Invivo 5, version 5.3 software. As shown in the picture, several landmarks (a total of five in this picture) that do not change after fracture were matched, and the CT image immediately after the operation and the follow-up CT image were superimposed. The blue impression is a postoperative CT image and the white impression is a follow-up CT image. **b-1** The measurement of the amount of displacement of the fractured segment. By adjusting the brightness and contrast of the postoperative CT and follow-up CT images, the margins of each bone fragment were well distinguished. After that, the highest points and lowest points of the fracture fragments of the two CT images were connected to each other, and the distance was displayed. **b-2** A magnified picture of the fracture site of the figure b-1. The amount of displacement of the fractured segment. The amount of displacement of the fractured segment was calculated by averaging the displacement of the highest points (a green number, 3.90 in this picture) and the lowest points (a red number, 3.17 in this picture) of the fracture. The displacement direction of the fractured segment. The displacement direction of the fractured segment was investigated by the direction of the line connecting the center point of the fracture fragment (blue arrow)

The patients were classified into the number of fixations. As a result, they were classified into 3 groups: 1-point fixation, 2-point fixation, and 3-point fixation. As in other studies, the three fixation points are the frontozygomatic suture (FZ suture), the infraorbital rim (IOR), and the zygomaticomaxillary buttress (ZMB) [1]. The amount and the direction of displacement of the fractured segment were analyzed in the coronal plane using the Invivo 5, version 5.3 software (Anatomage, San Jose, CA) with CBCT images taken before and after 1 month to 6 months follow-up. When measuring the displacement, the average of the displacement of the uppermost point and the lowermost point of the unfixed end of the fractured segment was calculated and compared. The direction of displacement was also investigated in which the midpoint of the line connecting the uppermost point and the lowermost point of the unfixed end of the fractured segment (Fig. 1).

Each time CT was taken, the Frankfort horizontal (FH) plane connecting the orbitale and the porion was parallel to the floor. Through this, when comparing the location of the fracture fragments after time, it was possible to match the location of the parts other than the fracture fragments. This method allows to compare the changes in the position of the fractured segment more accurately and more consistently.

All data were analyzed using SPSS software, version 19 (SPSS Inc, Chicago, IL). For statistical evaluation of the data of this study, the results of the normality test of 3 groups according to a fixed number were performed, They were nonparametric groups. So the Kruskal-Wallis *H* test was performed to compare the postoperative displacement according to the number of fractures.

## Results

The direction and the amount of movement of the fixed sites were compared in 38 patients (12 patients with 1-point fixation on ZM or FZ, 20 patients with 2-point fixation on ZM and IOR or ZM and FZ, and 6 patients with 3-point fixation on ZM, IOF, and FZ sites). The mean age of the patients was 48.48 ± 16.05 (mean ± standard deviation) years. The average period of the postoperative visit was 9.33 ± 8.26 weeks, and postoperative CT was taken between 2 weeks and 6 months (average 9.33 ± 8.26 weeks) after surgery, when the fracture was expected to be ossteointegrated, to check the amount of displacement and direction of the fractured segment (Table 1).

**Table 1:** Demographics of all patients

Characteristic	No.
Gender	
Male	31
Female	7
Average age (years)	48.58±16.05
Average follow-up CT period (weeks)	9.33±8.26
Fracture type	
No free segment	22
Free segment	16
Fixation type	
1-point	12
2-point	20
3-point	6

**Fig. 2:**
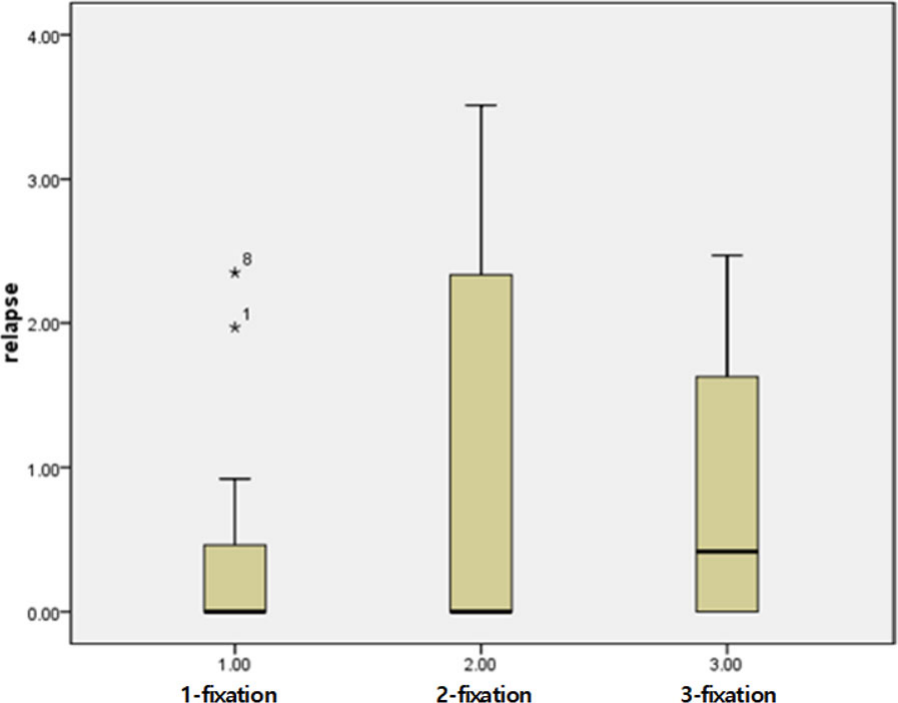
The amount of displacement according to the number of fixations. The asterisk and numbers 1 and 8 mean the patient number who are in the 1-fixation group

The positional change of the unfixed site showed an average of 0.75 ± 1.18 mm after 2~3 months postoperatively, and it showed mainly an inward change pattern. There was no statistically significant difference in the amount of the postoperative displacement according to the number of fixations (*p* = 0.574) (Fig. 2, Table 2). In other words, it can be interpreted that the amount of fixation did not make a difference in the postoperative stability of the fracture. Displacement of the distal end of the fractured segment was observed mainly inward. Among the 38 ZMC fractures, 12 cases of displacement were observed and 11 cases (91.67%) among those were displaced mainly inward. Of the two-point fixation, only one case where the ZM buttress and the FZ suture were fixed was observed downward displacement (Table 3).

**Table 2:** Displacement amount according to fixation numbers

	*n*	Mean (mm)	SD (mm)
1-point fixation	12	0.44	0.85
2-point fixation	20	0.92	1.38
3-point fixation	6	0.82	1.04
Total	38	0.75	1.18

**Table 3:** The direction of postoperative relapse of the fractured segment

Group (*n*)	Direction	No.
1-point (12)	M	2
	MD	1
	n	9
2-point (20)	MU	3
	M	1
	MD	1
	D	1
	n	14
3-point (6)	MU	1
	M	1
	MD	1
	n	3

*MU* mesial and upward, *M* mesial, *MD* mesial and downward, *n* no relapse

## Discussion

Open reduction after exposure of all buttresses and internal fixation using 3-point mini-plate fixation provides the best stability [1]. Rinehart et al. reported that in cadaver studies, 2-point or 3-point mini-plate fixation is sufficient to withstand mastication, but 1-point mini-plate fixation is not sufficient to stabilize the zygomatic fracture against mastication [5]. Davidson et al. reported that 3-point fixation with mini-plates or interosseous wires allows little displacement, and 2-point fixation with mini-plates provides clinically acceptable stability [3]. Unlike these studies, Chen et al. reported that high surgical stability can be obtained even with a 1-point fixation using a single vestibular approach [6]. Kim et al. also reported that in the case of ZMC fractures without compound fractures, 1-point fixation on ZMB provides sufficient stability [7].

In the case of comminuted fractures not included in this study, it is necessary to perform as many fixations as possible without causing controversy. However, a consensus has not been established for the fixation method of two or three simple ZMC fractures.

Since the ZMC, like the mandible, is not a region that continues to function such as opening or mastication, considering the stability after surgery, we have doubts about whether all fractures should be fixed. In particular, if more than two points are fixed, the skin around the eyeball can be scarred when the FZ suture is fixed, and the mini-plates can be touched because of the thin skin. The ZMB can be fixed by intraoral approach, and it has an esthetic advantage if it can be fixed only in one site. In addition to the fixation method, since many other factors, such as the experience of the operator, the degree of postoperative edema affect the postoperative stability, if similar stability can be obtained, it has clinical value in that performing a smaller number of fixations can reduce the operation time and makes less postoperative complications.

In our study, it was confirmed that the number of fixation sites did not make a difference in the postoperative stability of fractures. As a similar study to our study, a domestic paper analyzed 29 patients in 2012 reported that 1-point fixation was clinically sufficient for ZMC fracture without comminuted fractures.

If you know the direction of the postoperative displacement, which can means a kind of “relapse” of the fractures, reduction of ZMC fracture can be done considering relapse after surgery. Through this, it produces a stable surgical outcome in the long-term period. For this reason, this study investigated the direction of postoperative fractured segment displacement.

It is said that the most influential factor as the cause of postoperative displacement of the fractured segment is masticatory force, and the masseter muscle has the greatest role among masticatory forces. In this study, the direction of the displacement of the unfixed end of the fractured segment was observed mainly in the inward direction. Dal Santo et al. postoperative fractured segments are said that the masseter muscle continues to function postoperatively, and the fractured segments continue to receive downward force, thereby causing the fractures to be displaced [8]. When viewed from the front, the masseter muscle originating from the zygoma and touching the mandibular angle travels slightly inwardly and diagonally, so when the masseter muscle functions, the force acts in the inward direction on the zygoma fractured segment, and this will determine the direction of postoperative position change of the fractured segment.

The limitation of this study is that the number of patients investigated was small, and the period of visit after surgery was short. In the future, more patients with ZMC fractures should be examined to confirm the results of this study. Also, in this study, the displacement direction was investigated only in the coronal plane, but the displacement direction in the axial and sagittal planes should also be investigated. In addition to this, a comparative study on the postoperative stability of the cases of using an absorbent plate and non-absorbable plates is also considered meaningful.

## Conclusion

In the cases of open reduction and internal fixation of ZMC fracture, it was observed that the amount of fixation did not make a difference in the postoperative stability of the fracture. These results suggest that surgery can be less invasive by reducing the number of fixations. In addition, the fractured segment mainly changes in the inward direction after surgery, and this fact can be used as a reference for the reduction direction during surgery for a stable prognosis.

## Data Availability

The datasets generated and/or analyzed during the current study are not publicly available due [REASON WHY DATA ARE NOT PUBLIC] but are available from the corresponding author on reasonable request.

## Abbreviations

CT: Computed tomography
FH: Frankfort horizontal
FZ: Frontozygomatic
IOR: Infraorbital rim
ZA: Zygomatic arch
ZMB: Zygomaticomaxillary buttress
ZMC: Zygomaticomaxillary complex

## References

[1] Kim HJ, Bang KH, Park EJ, Cho YC, Sung IY, Son JH (2018). Evaluation of Postoperative Stability After Open Reduction and Internal Fixation of Zygomaticomaxillary Complex Fractures Using Cone Beam Computed Tomography Analysis. J Craniofac Surg.

[2] Kim ST, Go DH, Jung JH, Cha HE, Woo JH, Kang IG (2011). Comparison of 1-point fixation with 2-point fixation in treating tripod fractures of the zygoma. J Oral Maxillofac Surg.

[3] Davidson J, Nickerson D, Nickerson B (1990). Zygomatic fractures: comparison of methods of internal fixation. Plast Reconstr Surg.

[4] O'Hara DE, DelVecchio DA, Bartlett SP, Whitaker LA (1996). The Role of Microfixation in Malar Fractures: A Quantitative Biophysical Study. Plast Reconstr Surg.

[5] Rinehart GC, Marsh JL, Hemmer KM, Bresina S (1989). Internal fixation of malar fractures: an experimental biophysical study. Plast Reconstr Surg.

[6] Chen CH, Mao SH, Shyu VB, Chen CT (2015). Single buccal sulcus approach with fluoroscan assistance for the management of simple zygomatic fractures. Ann Plast Surg.

[7] Kim JH, Lee JH, Hong SM, Park CH (2012). The effectiveness of 1-point fixation for zygomaticomaxillary complex fractures. Arch Otolaryngol Head Neck Surg.

[8] Dal Santo F, Ellis E, Throckmorton GS (1992). The effects of zygomatic complex fracture on masseteric muscle force. J Oral Maxillofac Surg.

